# Brain Active Areas Associated with a Mental Arithmetic Task: An eLORETA Study

**DOI:** 10.3390/bioengineering10121388

**Published:** 2023-12-03

**Authors:** Serena Dattola, Lilla Bonanno, Augusto Ielo, Angelica Quercia, Angelo Quartarone, Fabio La Foresta

**Affiliations:** 1 IRCCS Centro Neurolesi Bonino-Pulejo, Via Palermo c/da Casazza, SS. 113, 98124 Messina, Italy; serena.dattola@irccsme.it (S.D.); lilla.bonanno@irccsme.it (L.B.); angelo.quartarone@irccsme.it (A.Q.); 2 Department of Biomedical, Dental, Morphological and Functional Imaging Sciences, University of Messina, 98122 Messina, Italy; angelica.quercia@unime.it; 3 DICEAM Department, Mediterranea University of Reggio Calabria, Via Graziella Feo di Vito, 89060 Reggio Calabria, Italy; fabio.laforesta@unirc.it

**Keywords:** mental arithmetic task, EEG, eLORETA, limbic lobe, rehabilitation

## Abstract

The neural underpinnings of mental calculation, the fundamentals of arithmetic representations and processes, and the development of arithmetic abilities have been explored by researchers over the years. In the present work, we report a study that analyzes the brain-activated areas of a group of 35 healthy subjects (9 males, 26 females, mean age ± SD = 18.23 ± 2.20 years) who performed a serial subtraction arithmetic task. In contrast to most of the studies in the literature based on fMRI, we performed the brain active source reconstruction starting from EEG signals by means of the eLORETA method. In particular, the subjects were classified as bad counters or good counters, according to the results of the task, and the brain activity of the two groups was compared. The results were statistically significant only in the beta band, revealing that the left limbic lobe was found to be more active in people showing better performance. The limbic lobe is involved in visuospatial processing, memory, arithmetic fact retrieval, and emotions. However, the role of the limbic lobe in mental arithmetic has been barely explored, so these interesting findings could represent a starting point for future in-depth analyses. Since there is evidence in the literature that the motor system is affected by the execution of arithmetic tasks, a more extensive knowledge of the brain activation associated with arithmetic tasks could be exploited not only for the assessment of mathematical skills but also in the evaluation of motor impairments and, consequently, in rehabilitation for motor disorders.

## 1. Introduction

The study of human cognition and cognitive performance has recently become more popular and significant. Cognitive training programs maintain and improve cognitive functions, like attention, reasoning, memory, and learning [1,2]. These cognitive abilities need to be effective to successfully complete a task. Moreover, cognitive training can help to improve attentional control and can be used as a therapeutic and rehabilitation tool in clinical populations to alleviate not only cognitive symptoms but also motor deficits; as a result, functional benefits, such as improved balance and postural control, can be expected after the cognitive training [3]. It is well known that mental tasks are a particularly effective form of brain stimulation, useful for its development. Retrieving reality, remembering things, sequencing things, making decisions, and performing calculations are all mental tasks. Calculations, mental comparisons, quantity identification, and other sophisticated processes are all part of arithmetic. The neurological foundation of mental calculation, the underlying principles of arithmetic representations and processes, and the evolution of arithmetic skills are some research matters in this field [4,5,6]. Mental arithmetic tasks have long been a subject of study within the scientific literature, particularly in the fields of cognitive psychology and neuroscience. These tasks involve the ability to perform complex mathematical calculations mentally, without the use of external aids such as calculators or pen and paper. The study of mental arithmetic sheds light on various aspects of human cognition, including working memory, attention, problem-solving, and the neural mechanisms underlying mathematical processing [7,8,9]. One of the key areas of interest in the scientific literature is the cognitive processes involved in mental arithmetic. Researchers have explored how individuals manipulate numbers in their minds, examining the strategies and techniques they employ [10,11,12]. Mental arithmetic often relies on working memory, the cognitive system responsible for temporarily holding and manipulating information [13,14]. Studies have shown that individuals with strong working memory capacity tend to perform better on mental arithmetic tasks, suggesting a close relationship between this cognitive resource and mathematical ability [15,16]. Additionally, researchers have investigated the role of attention and executive functions in mental arithmetic, highlighting the importance of sustained attention and inhibitory control when solving complex mathematical problems mentally [17,18]. Another significant aspect of mental arithmetic research is the development of mathematical skills in children and adults. Studies have examined how mental arithmetic abilities evolve throughout the lifespan, from early childhood to old age [19,20,21,22]. This research has provided insights into the stages of mathematical development and the impact of educational interventions on mental arithmetic proficiency. Moreover, investigations into developmental dyscalculia, a specific learning disorder related to mathematical difficulties, have shed light on the neural and cognitive underpinnings of this condition [23].

Over the years, brain imaging has been extensively used to investigate the brain’s reaction to various cognitive activities, including numerical and mathematical processing [24]. Neuroimaging techniques include, among others, magnetoencephalography (MEG), positron emission tomography (PET), functional magnetic resonance imaging (fMRI), and electroencephalography (EEG). In particular, EEG is a non-invasive technique that allows for the examination of temporal and spatial features of brain activity [25]. Its relatively low cost and portability have made it the most widely used imaging method in both clinical and research settings. Over time, researchers have exploited the potentiality of EEG for investigating the brain’s behavior during the execution of arithmetic tasks. Several researchers proposed different methods based on neural networks for discriminating arithmetic task conditions from resting conditions. Kim et al. [26] analyzed the application of the EEG microstate to evaluate the performance during a mental arithmetic task. The results revealed that the highest mean Area Under Curve (AUC) using eleven microstate features selected by recursive feature elimination was 0.831, showing a high capability in distinguishing good performers from bad ones. The system proposed by Maghsoudi and colleagues [27] achieved an accuracy of 89% in discriminating between mental arithmetic tasks and resting state using effective connectivity quantified with the Generalized Partial Directed Coherence (GPDC) and feature selection via concave minimization method. Hoda Edris et al. [28] extracted several geometric features using the Poincaré diagram and performed classification to differentiate mental tasks from rest mode by means of an artificial neural network (ANN). The results showed that, if selected EEG channels were considered, accuracy could achieve 100%. Dutta et al. [29] described a new method for feature extraction based on the combination of multivariate empirical mode decomposition (MEMD) and multivariate auto-regressive (MVAR) model. The classification performed by the Least squares support vector machine (LS-SVM) using the polynomial kernel function showed an average classification accuracy of 94.43% in differentiating the baseline from the mental arithmetic task. In [30], the auto regression (AR) model and the wavelet transform were used for extracting the features, which were employed for testing k-nearest neighbor (K-NN) and SVM classifiers. The highest accuracy in discriminating between rest and task conditions was 92% for the following combinations: wavelet features/SVM classifier, all features/SVM classifier, and AR coefficients/k-NN classifier. In [31] the authors introduced a new method for multifractal analysis of EEG signals named generalized Higuchi fractal dimension spectrum (GHFDS). AR features, statistical features, power spectrum density (PSD) features, and GHFDS features were extracted and analyzed. The results showed that the combination of all features led to a higher mean classification accuracy (97.87%). Chatterjee et al. [32] proposed a novel method to summarize the window-level features and consequently develop the descriptor at the signal level. Several classifiers were tested using the proposed method to discriminate good counters from bad counters. The results revealed that the Gaussian naıve Bayes classifier outperformed the other classifiers (mean accuracy 85%). The study in [33] analyzed the brain network connectivity parameters in a resting state and during the execution of an arithmetic task for all EEG frequency bands. The subjects were divided into successful and unsuccessful counters according to the correctness of calculations. The results indicated that successful subjects showed higher connectivity in the rest condition for most EEG bands, above all in the gamma band, and for task condition in the gamma band. Kitaura and colleagues [34], using the sLORETA method, found a significantly increased activity in medial prefrontal areas and decreased activity in the left parietal lobe for the theta band during the arithmetic task as compared to the resting state. Moreover, the results showed a decreased activity in parietal-occipital regions for the alpha1 band. In addition, connectivity within the right hemisphere decreased during the task, whereas connections in the left hemisphere increased.

The aforementioned EEG-based studies performed analyses concerning the differences between resting and mental task conditions. According to the literature, the reconstruction of the brain’s active areas during an arithmetic task has been mainly evaluated using fMRI [35], whereas, in our study, this was achieved by using the EEG signals. In particular, we used the eLORETA method to compare the brain activity at the source level of two groups of healthy subjects, classified as bad counters or good counters, according to the results of the mental arithmetic task they performed.

## 2. Materials and Methods

### 2.1. Dataset

The dataset tested in this study is publicly available, and it can be downloaded at the following link: https://physionet.org/content/eegmat/1.0.0/ (accessed on 3 May 2023) [36,37]. The EEGs were collected by Neurocom monopolar EEG 23-channel system (Ukraine, XAI-MEDICA), using 19 electrodes according to the international 10–20 system: Fp1, Fp2, F3, F4, Fz, F7, F8, C3, C4, Cz, P3, P4, Pz, O1, O2, T3, T4, T5, and T6, with linked earlobe reference. The electrode impedance was kept below 5 kΩ, and the sampling rate was 500 Hz. The EEGs were high-pass filtered at 0.5 Hz, low-pass filtered at 45 Hz cut-off frequency, and a power line notch filter (50 Hz) was applied. Artifacts were removed using the Independent Component Analysis (ICA). The database includes 36 subjects (9 males, 27 females, mean age ± SD = 18.25 ± 2.17). Participants were included in the database if they had typical or appropriately corrected visual acuity or regular color vision and showed no clinical signs of mental or cognitive dysfunction or difficulties in verbal or non-verbal learning. Exclusion criteria encompassed the utilization of psychoactive medications, substance addiction, and the presence of psychiatric or neurological issues. Subject 31 was not considered because the recordings were different in duration, so our study involved 35 subjects (9 males, 26 females, mean age ± SD = 18.23 ± 2.20). The demographic data of the subjects taken into consideration are reported in Table 1 and in Table S1. The arithmetic task consisted of the serial subtraction of two numbers for 4 min. A minuend (4-digit) and a subtrahend (2-digit) were verbally communicated, and the participants were asked to perform a mental count without speaking or moving fingers. At the end of the task, each subject shared the result of the calculation. If the result did not deviate by more than 20% from the correct one, then the participants were considered successful in the task. Finally, the subjects were grouped into good and bad counters. The “Bad” counters (group “B”, 10 subjects, mean number of operations per 4 min = 7, SD = 3.6) completed the task with greater difficulty; the “Good” counters performed the task easily (group “G”, 25 subjects, mean number of operations per 4 min = 21, SD = 7.4). EEGs were recorded in a resting state with eyes closed for 3 min and during the first minute of the execution of the arithmetic task. All the EEG recordings (rest and task) were re-referenced to a common average reference montage and divided into artifact-free, non-overlapping epochs of 1500 samples (3 s) using MATLAB (R2022a).

### 2.2. Brain Source Localization with eLORETA

In this study, the brain activity of bad and good counters was computed starting from the EEG signals. In order to find the location of the active sources that are responsible for the measured EEG data, it is necessary to solve the EEG inverse problem [38]. To this end, we used eLORETA, which is the latest algorithm of the LORETA family methods [39,40]. This method provides the values of the standardized current density for each source of the brain volume (gray matter and hippocampus), which consists of 6239 voxels at 5 mm spatial resolution, using the MNI152 template [41]. Each brain source is placed on a voxel and is represented by a current density vector. The validity of LORETA localization results has been supported by cross-validation studies that simultaneously used fMRI [42,43,44,45,46]. As in sLORETA [47], in eLORETA the estimation of current density employs the minimum norm solution, which is then standardized by its variance. Notably, in contrast to the Dale method [48], this variance computation takes into account not only the noise arising from the EEG measurements but also the biological noise originating from the actual sources, contributing to a more refined electric potential variance estimation. Moreover, differently from sLORETA, eLORETA introduces a weight matrix designed to consider deeper sources in a more effective way, leading to a further reduction of the localization error. Indeed, eLORETA demonstrates improved capability in suppressing less significant sources and generates less blurred images compared to sLORETA [49].

**Figure 1:**
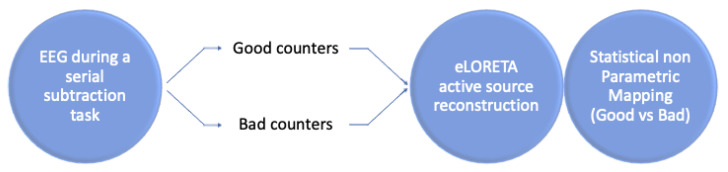
Flow diagram of the experimental procedure.

In this work, the analysis was performed by means of the free LORETA-KEY software (v20221229). The image of the activated brain areas provided by the software represents the power current density of each voxel. This image is obtained by averaging the images computed for each time sample. The power current density was calculated by eLORETA for each subject for the following frequency bands: delta (0.5–4 Hz), theta (4–8 Hz), alpha (8–13 Hz), beta (13–30 Hz), gamma (30-45 Hz). First, we considered the voxel-by-voxel difference of the power current density values between the task and the corresponding rest condition (baseline) for each subject. In this way, we quantified the activation of each subject’s brain areas during the execution of the arithmetic task. The eLORETA images for each subject are available in Table S2. Then, a statistical comparison between the eLORETA images of “G” and “B” groups was carried out. The scheme illustrating the experimental procedure is shown in Figure 1.

### 2.3. Statistical Analysis

A statistical analysis was performed to evaluate the differences in the power current density between the “G” and “B” groups for each frequency band. The statistical tests used in this work are based on the Statistical non-Parametric Mapping (SnPM) methodology implemented in the LORETA-KEY software. SnPM is a robust methodology widely used in neuroimaging studies for analyzing complex datasets. Unlike parametric methods, SnPM does not require specific population parameter estimations, making it well-suited for various data distributions, be it Gaussian or non-Gaussian [50]. SnPM utilizes permutation-based techniques, randomly reassigning conditions to produce a null distribution, allowing for robust statistical inference without relying on underlying assumptions. Moreover, SnPM provides correction procedures for multiple comparisons, ensuring rigorous control over false positives. Its versatility and resilience against outliers make SnPM a valuable tool for exploring brain activity patterns and differences. In fact, the robustness of SnPM has been exemplified in neuroimaging studies, with several well-documented references attesting to its effectiveness in this context [51,52]. Moreover, the study reported in [53] has shown that both parametric and non-parametric statistics are valid tools when applied to LORETA. In our work, we conducted voxel-by-voxel t-statistic tests with 5000 randomizations on eLORETA log-transformed power current density. The tool also performed the correction of critical thresholds and p-values for multiple comparisons. We set the significance level at 5%, so the group differences were considered statistically significant when *p*-corrected < 0.05. This parameter ensures that the reported differences in brain activity between the “G” and “B” groups are not due to chance but are indeed indicative of true disparities in neural engagement during the mental arithmetic task.

## 3. Results

**Figure 2:**
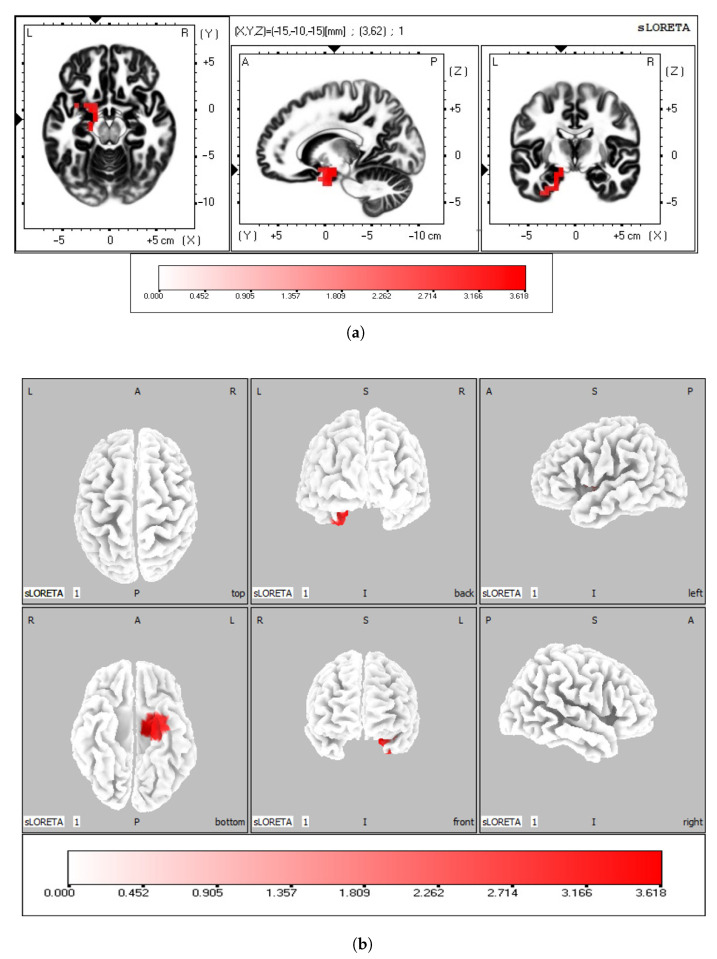
Differences in the power current density between “G” and “B” groups. (**a**) slice view, and (**b**) 3D brain cortex from six different viewpoints, with the corresponding color scale bar. Colored areas represent the statistically significant suprathreshold voxels.

To assess the active brain areas, the power current density of each subject was computed using eLORETA. This analysis was performed separately for each frequency band, allowing for a comprehensive examination of how brain activity varies across different neural rhythms. The comparison between the “G” and “B” groups was performed to detect possible differences in brain activity. The statistical analysis revealed that significant differences were found only in the beta band (t-log threshold = 3.094, *p*-corrected = 0.0136, one-tailed). In particular, the good counters showed higher activation in the left limbic lobe as compared to the bad counters. The SnPm tool provided the images representing the difference in the power current density of each voxel between the “G” and “B” groups in the neuroanatomic MNI space. Figure 2 shows the statistically significant voxels that were more active for the “G” group as compared to the “B” group in the beta band. The maximum difference was found in the parahippocampal gyrus (BA 28, MNI coordinates: x = −15, y = −10, z = −15). Significant differences were found in the following brain regions: the uncus (t-log threshold = 3.488, *p*-corrected = 0.0178, one-tailed), the parahippocampal gyrus (t-log threshold = 3.618, *p*-corrected = 0.0134, one-tailed), the insula (t-log threshold = 3.295, *p*-corrected = 0.0178, one-tailed), the inferior temporal gyrus (t-log threshold = 3.280, *p*-corrected = 0.0178, one-tailed), the subcallosal gyrus (t-log threshold = 3.272, *p*-corrected = 0.0178, one-tailed), the sub-gyral (t-log threshold = 3.110, *p*-corrected = 0.0228, one-tailed), and the superior temporal gyrus (t-log threshold = 3.127, *p*-corrected = 0.0228, one-tailed). For a more comprehensive overview, Table 2 reports the number of statistically significant suprathreshold voxels and the brain structure they belong to. Table 3 shows the MNI coordinates of the suprathreshold voxel whose value corresponds to the maximum difference between “G” and “B” for each brain area. Most of the voxels are part of the limbic lobe. As for the other brain rhythms, no significant differences were found between good and bad counters for delta, theta, alpha, and gamma bands.

## 4. Discussion

Mental arithmetic tasks cover a rich and varied area of study in the scientific literature, encompassing cognitive, developmental, neuroscientific, and practical aspects. Researchers have made significant strides in understanding the cognitive processes involved in mental arithmetic, the developmental trajectory of mathematical abilities, the neural substrates of calculation, and the real-world implications of mental arithmetic skills. Mental arithmetic tasks represent a widely employed tool for exploring brain activity during the execution of calculations. As for the brain areas associated with mathematical cognition, it is well known that bilateral parietal and frontal lobes are the most activated areas during arithmetic tasks [19,54]. In particular, a recent fMRI meta-analysis examined 31 studies, revealing that the brain regions involved in mental arithmetic are the following (from largest to smallest): the left inferior parietal lobule (hIP3), right precuneus, left inferior frontal gyrus, left superior frontal gyrus, left insula, right insula, right middle frontal gyrus, left middle frontal gyrus, and right subgyral [35]. In this work, we evaluated the difference in brain activation between subjects classified as bad and good counters according to their performance in an arithmetic task. In particular, the good counters revealed greater activation in comparison to the bad counters. Most of the significantly different voxels belong to the left limbic lobe, specifically to the following brain areas: uncus, parahippocampal gyrus, and insula (Table 2). To the best of our knowledge, the role of the limbic lobe in mental arithmetic has been barely explored. The parahippocampal gyrus is associated with several cognitive functions, such as visuospatial processing and memory [55]. Recent studies have shown that this area is involved in arithmetic fact retrieval [56,57]. The results reported in [58] showed that the parahippocampal gyrus was the most activated region during the calculation tasks. Moreover, the meta-analysis carried out in [59] showed that performing subtraction tasks produces significant activity in the insular cortex. According to previous studies, insula can be implicated in intrinsically motivated behaviors [60,61]. We could hypothesize that the good counters are more motivated in the execution of the task than the bad counters. The uncus is a limbic area that is supposed to be involved in emotions and memory processing [62,63]. Moreover, the results of the current study revealed that there is a significant difference between the two groups only in the beta band. Recently, the beta band has been found to be involved in emotion and long-term memory tasks [64]. However, the beta frequency band is mainly considered the default rhythm of the sensorimotor system, tightly associated with motor processes [65,66,67]. Beta oscillations are found in almost all structures involved in motor processes, including muscles, dorsal and basal ganglia, and cortex. Indeed, motor actions modulate beta oscillations. Specifically, during motor planning and execution, a decrement of beta band power occurs (event-related desynchronization, ERD); conversely, an increase in beta power occurs at the end of the movement (event-related synchronization, ERS). Moreover, the enhancement of beta power coherence correlates with active processes in motor control [68]. Interestingly, there is evidence in the literature that the motor system is affected by the execution of arithmetic tasks. Pavao et al. [69] investigated the effects of dual-tasking (motor task concurrent to an arithmetic task) under different conditions in three groups of subjects: children, adolescents, and young adults. The findings showed that the execution of a concurrent task altered postural stability in the participants, according to the difficulty of the tasks. In particular, children exhibited lower dual-task costs (defined as performance differences between dual and single-task trials), measured by area, during easy cognitive tasks compared to young adults. Across all cognitive conditions, dual-task costs were reduced when participants had a narrow base of support as opposed to a standard one. Additionally, regardless of the tested bases of support, dual-task costs based on velocity were lower for more difficult cognitive tasks than for those classified as easy. In older adults, static postural control can be improved by the execution of concurrent arithmetic tasks [70]. A meta-analysis reported in [71] revealed that dual tasks negatively affect gait performance in Parkinson’s Disease. Bensoussan and colleagues [72] found that a mental arithmetic task can impact motor neuron activity, which may affect the assessment of motor impairments and the rehabilitation of movement disorders. In [73], the authors demonstrated that hemiplegic patients of different ages with chronic stroke experienced alterations in their postural control mechanisms when undertaking a simple arithmetic task, providing a useful tool to assess postural control in these patients. Vuillerme and colleagues [74] found that challenging mental arithmetic tasks reduced center of foot pressure (COP) displacements during bipedal standing, likely due to increased stiffness and decreased exploratory behaviors in the short term. In [75], the authors investigated the impact of a dual task on postural stiffness by utilizing a serial subtraction task and found that it increased sway amplitude in community-dwelling older adults. Future research may explore how arithmetic tasks affect motor neuron activity, enhancing movement disorders evaluation and shaping cognitive-motor intervention strategies in rehabilitation.

## 5. Limitations

The main limitation of this work lies in the imbalance of subjects across groups, which requires a future investigation with a larger and more evenly distributed database. The current dataset’s uneven distribution can potentially influence the robustness and generalizability of the findings. Moreover, the use of a standard EEG system with only 19 electrodes may restrict the spatial resolution of the recorded signals. To address this limitation, further developments of this research should include signals acquired by HD-EEG systems. Integrating HD-EEG technology can significantly improve spatial resolution, providing a more comprehensive and detailed exploration of the activated brain areas during the mental arithmetic task. Regarding the methodology used to solve the EEG inverse problem, only a single method was employed in this study. Although the eLORETA algorithm is one of the most established methods for solving the EEG inverse problem, future research would include a comparison with other state-of-the-art methods.

## 6. Conclusions

In contrast to the previous research based on fMRI, the novel aspect of this work is the application of the eLORETA method to study the performance during an arithmetic task. The results indicate higher activity in the left limbic lobe in subjects who demonstrated a better ability to complete the arithmetic task. These findings hint at the potential role of the limbic lobe in arithmetic task performance. This outcome is particularly intriguing given the limbic lobe’s established roles in memory and emotional processing, suggesting a complex cognitive process underlying arithmetic abilities. Given the limited research concerning the connection between this brain area and arithmetic skills, this preliminary outcome offers a compelling foundation for further in-depth analyses focused on the involvement of the limbic lobe in the performance of an arithmetic task. Further, these results pave the way for exploring the integration of cognitive functions in complex tasks. Future research could investigate how different brain regions coordinate and communicate during such tasks, potentially revealing new insights into the brain’s adaptive mechanisms for handling cognitive challenges. Future analyses should also include EEG signals recorded by high-density systems to achieve greater spatial resolution. This advancement would contribute to a deeper understanding of the neural dynamics underpinning cognitive processes.

Moreover, the interaction between cognitive and motor functions could lead to novel approaches in neurorehabilitation. For instance, incorporating arithmetic tasks in motor rehabilitation programs could leverage cognitive-motor interaction, possibly enhancing recovery outcomes for patients with motor disorders. Such interdisciplinary applications highlight the broad impact of understanding brain function in specific cognitive tasks.

## Figures and Tables

**Table 1:** Description of the study population.

	All	Male	Female
Participants	35	9 (25.7%)	26 (74.3%)
Age (years)	18.23 ± 2.20	19.67 ± 3.46	17.73 ± 1.31
Occupation			
Student	35	9 (25.7%)	26 (74.3%)
Count quality			
Good	25	6 (24%)	19 (76%)
Bad	10	3 (30%)	7 (70%)

**Table 2:** List of the statistically significant suprathreshold voxels and their corresponding brain areas in the beta band.

Brodmann Areas	Structure	Lobe	Number of Voxels	*p*-Values
20, 28, 34, 36, 38	Uncus	Limbic	29	0.0178
28, 34, 35, 36	Parahippocampal gyrus	Limbic	23	0.0134
13	Insula	Sub-Lobar	12	0.0178
20	Inferior Temporal Gyrus	Temporal, Limbic	4	0.0178
34	Subcallosal Gyrus	Frontal	3	0.0178
13, 21	Sub-Gyral	Temporal	2	0.0228
38	Superior Temporal Gyrus	Temporal	2	0.0228

**Table 3:** MNI coordinates of the suprathreshold voxel corresponding to the maximum difference value for each brain area in the beta band.

Structure	Brodmann Area	MNI Coordinates		
		**x**	**y**	**z**
Uncus	34	−15	−5	−25
Parahippocampal gyrus	28	−15	−10	−15
Insula	13	−35	5	15
Inferior Temporal Gyrus	20	−30	−5	−45
Subcallosal Gyrus	34	−25	5	−15
Sub-Gyral	13	−40	0	−10
Superior Temporal Gyrus	38	−35	5	−15

## Data Availability

Raw data supporting the conclusions of this article will be made available by the authors upon request.

## Supplementary Materials

The following supporting information can be downloaded at: https://www.mdpi.com/article/10.3390/bioengineering10121388/s1, Table S1: Demographic data of the recruited subjects; Table S2: Difference of the power current density values between the task and the corresponding rest condition for each subject.
