# Supplementary material for: Brain Active Areas Associated with a Mental Arithmetic Task: An eLORETA Study
Source: Bioengineering (Basel). 2023 Dec 3;10(12):1388. doi: 10.3390/bioengineering10121388 (PMC10740510; doi:10.3390/bioengineering10121388)
Supplement: Supplementary file 1 [file bioengineering-10-01388-s001.zip › bioengineering-2673977-supplementary.pdf]

bioengineering

MDPI

Article

Brain Active Areas Associated with a Mental Arithmetic Task: An eLORETA Study

Serena Dattola [1] , Lilla Bonanno [1], Augusto Ielo [1,*], Angelica Quercia [2] and Angelo Quartarone [1] and Fabio La Foresta [3]

1. IRCCS Centro Neurolesi Bonino-Pulejo, Via Palermo c/da Casazza, SS. 113, 98124 Messina, Italy; serena.dattola@irccsme.it (S.D.); lilla.bonanno@irccsme.it (L.B.); angelo.quartarone@irccsme.it (A.Q.)
2. Department of Biomedical, Dental, Morphological and Functional Imaging Sciences, University of Messina, 98122 Messina, Italy; angelica.quercia@unime.it
3. DICEAM Department, Mediterranea University of Reggio Calabria, Via Graziella Feo di Vito, 89060 Reggio Calabria, Italy; fabio.laforesta@unirc.it

* Correspondence: Correspondence: augusto.ielo@irccsme.it

Supplementary Materials

Table S1. Demographic data of the recruited subjects.

Subject ID	Age	Gender	Occupation	Number of subtractions	Count quality
Subject00	21	F	Student	9.70	B
Subject01	18	F	Student	29.35	G
Subject02	19	F	Student	12.88	G
Subject03	17	F	Student	31	G
Subject04	17	F	Student	8.60	B
Subject05	16	F	Student	20.71	G
Subject06	18	M	Student	4.35	B
Subject07	18	F	Student	13.38	G
Subject08	26	M	Student	18.24	G
Subject09	16	F	Student	7	B
Subject10	17	F	Student	1	B
Subject11	18	F	Student	26	G
Subject12	17	F	Student	26.36	G
Subject13	24	M	Student	34	G
Subject14	17	F	Student	9	B
Subject15	17	F	Student	22.18	G
Subject16	17	F	Student	11.59	G
Subject17	17	F	Student	28.70	G
Subject18	17	F	Student	20	G
Subject19	22	M	Student	7.06	B
Subject20	17	F	Student	15.41	G
Subject21	20	F	Student	1	B
Subject22	19	F	Student	4.47	B
Subject23	16	F	Student	27.47	G
Subject24	17	M	Student	14.76	G
Subject25	17	M	Student	30.53	G

Subject26	17	F	Student	13.59	G
Subject27	19	F	Student	34.59	G
Subject28	19	F	Student	27	G
Subject29	19	M	Student	16.59	G
Subject30	17	M	Student	10	B
Subject32	20	F	Student	13	G
Subject33	17	M	Student	21.47	G
Subject34	18	F	Student	31	G
Subject35	17	F	Student	12.18	G

B stands for "Bad counters", G stands for "Good counters".

Table S2. Difference of the power current density values between the task and the corresponding rest condition for each subject.

**Subject ID**	**Max power current density value**	**eLORETA images**
Subject00	Value= 1,91E+0<br>(X= 15 , Y= 65 , Z= -15) (MNI coords)<br><br>Brodmann area 11<br>Superior Frontal Gyrus<br>Frontal Lobe	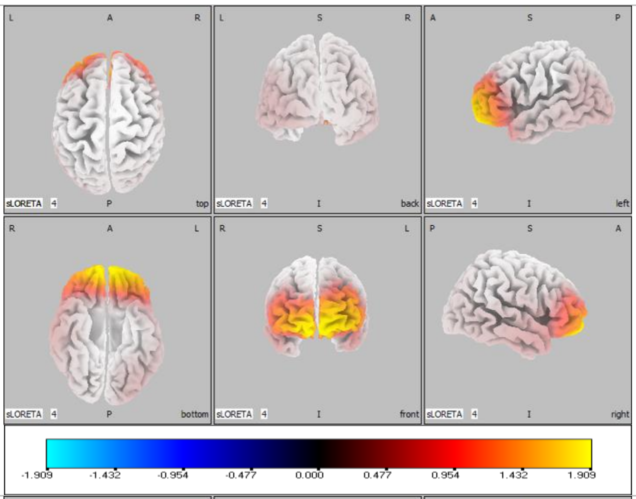
Subject01	Value= 4,33E+0<br>(X= 30 , Y= -80 , Z= 45) (MNI coords)<br><br>Brodmann area 7<br>Superior Parietal Lobule<br>Parietal Lobe	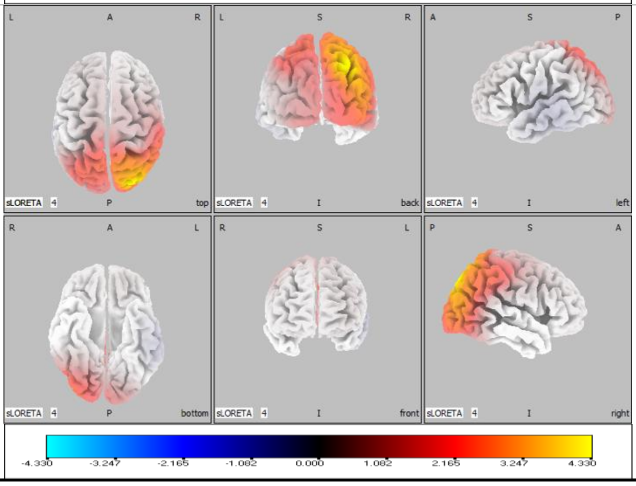

Subject02	Value= -1,92E+0<br>(X= -40 , Y= 55 , Z= 5) (MNI coords)<br><br>Brodmann area 10<br>Inferior Frontal Gyrus<br>Frontal Lobe	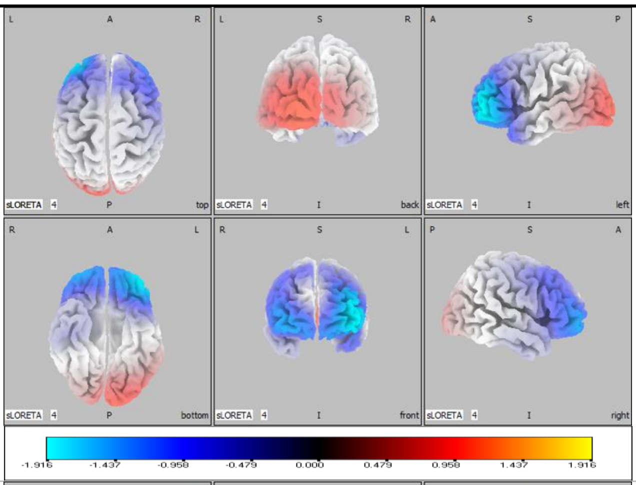
Subject03	Value= 3,37E-1<br>(X= 45 , Y= -85 , Z= -10) (MNI coords)<br><br>Brodmann area 19<br>Inferior Occipital Gyrus<br>Occipital Lobe	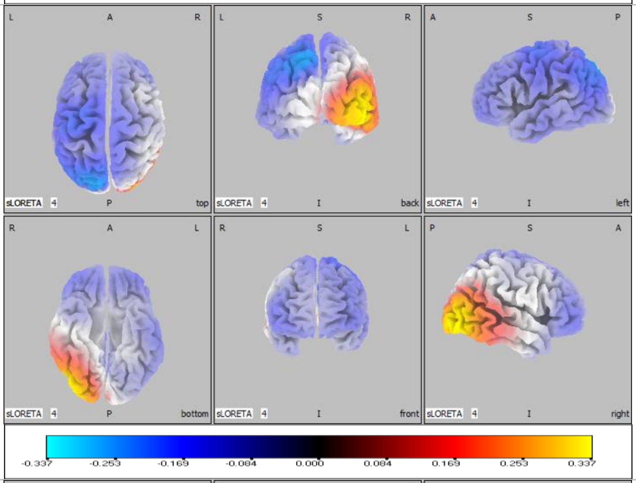
Subject04	Value= -8,57E-1<br>(X= -55 , Y= -75 , Z= 10) (MNI coords)<br><br>Brodmann area 39<br>Middle Temporal Gyrus<br>Temporal Lobe	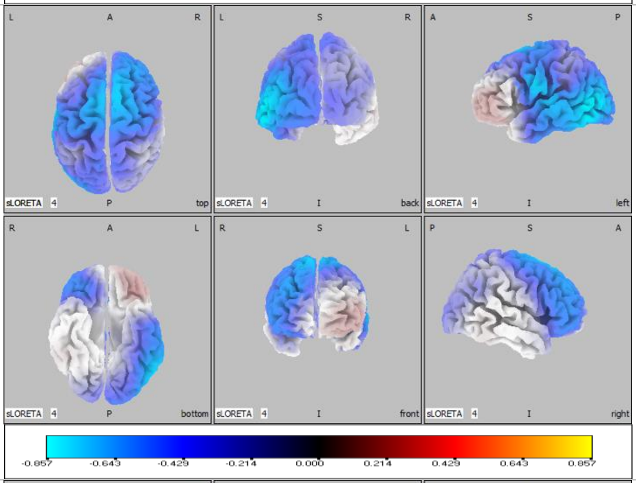
Subject05	Value= 2,96E-1<br>(X= 25 , Y= -65 , Z= 65) (MNI coords)<br><br>Brodmann area 7<br>Superior Parietal Lobule<br>Parietal Lobe	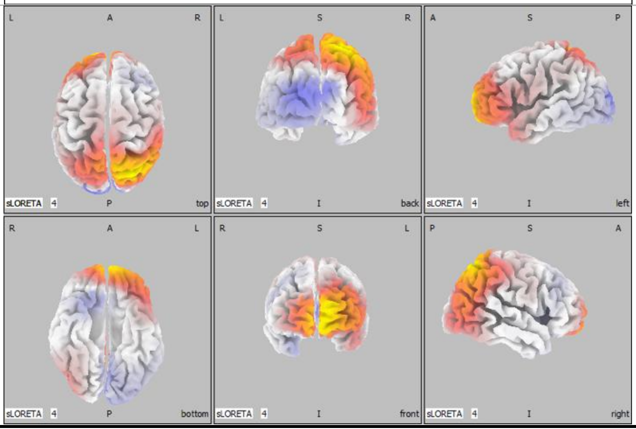

		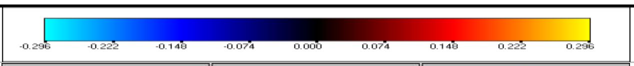
Subject06	Value= 2,62E-1<br>(X= -35 , Y= 55 , Z= 20) (MNI coords)<br><br>Brodmann area 10<br>Superior Frontal Gyrus<br>Frontal Lobe	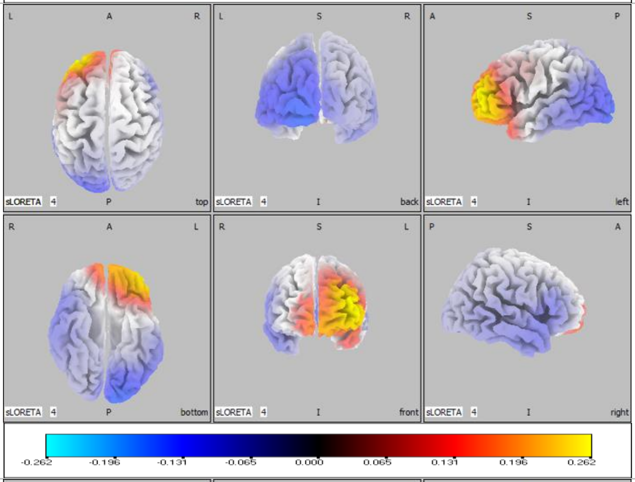
Subject07	Value= 8,62E-1<br>(X= 20 , Y= 65 , Z= -15) (MNI coords)<br><br>Brodmann area 11<br>Superior Frontal Gyrus<br>Frontal Lobe	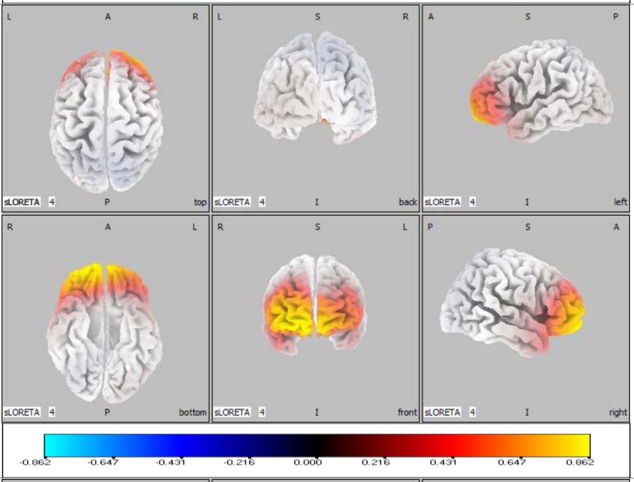
Subject08	Value= 2,02E-1<br>(X= 20 , Y= -100 , Z= 0) (MNI coords)<br><br>Brodmann area 18<br>Middle Occipital Gyrus<br>Occipital Lobe	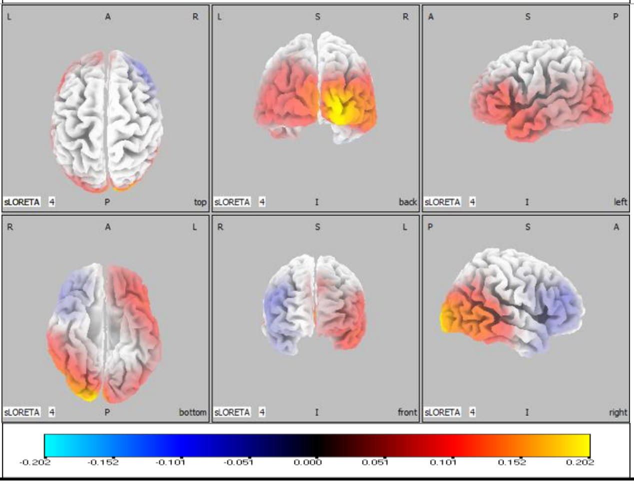

Subject09	Value= 5,11E-1<br>(X= -50 , Y= -55 , Z= 50) (MNI coords)<br><br>Brodmann area 40<br>Inferior Parietal Lobule<br>Parietal Lobe	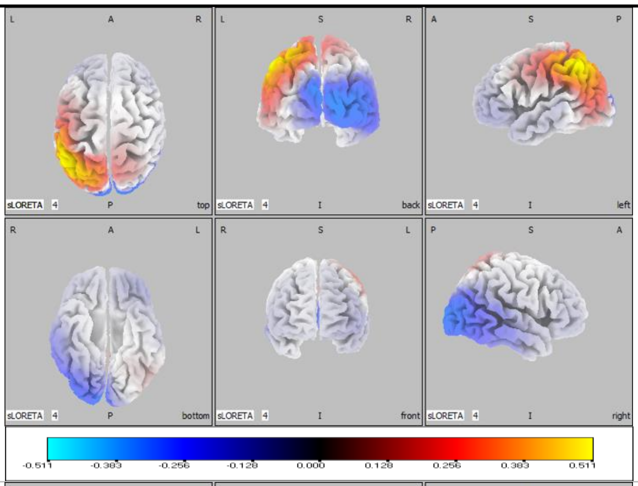
Subject10	Value= 6,87E-1<br>(X= 5 , Y= 65 , Z= -15) (MNI coords)<br><br>Brodmann area 11<br>Medial Frontal Gyrus<br>Frontal Lobe	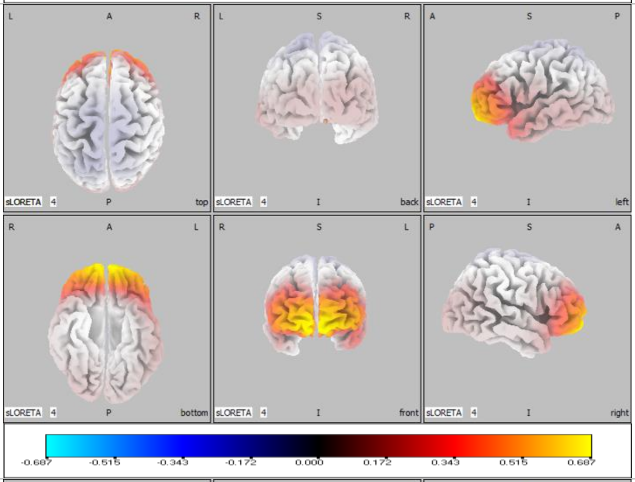
Subject11	Value= 1,03E-1<br>(X= 45 , Y= 20 , Z= -35) (MNI coords)<br><br>Brodmann area 38<br>Superior Temporal Gyrus<br>Temporal Lobe	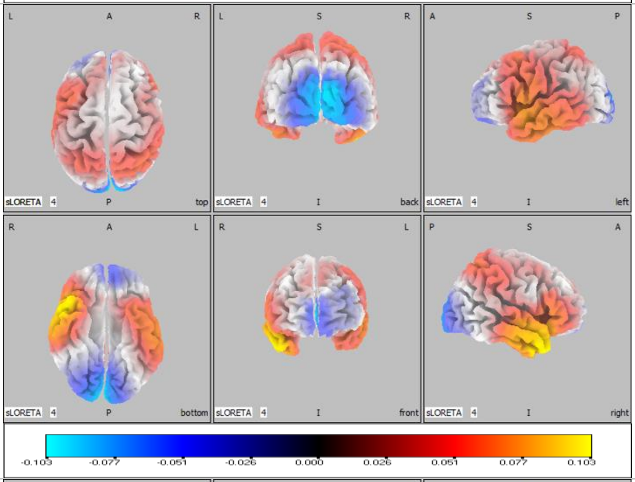
Subject12	Value= -9,82E+0<br>(X= 70 , Y= -20 , Z= -10) (MNI coords)<br><br>Brodmann area 21<br>Middle Temporal Gyrus<br>Temporal Lobe	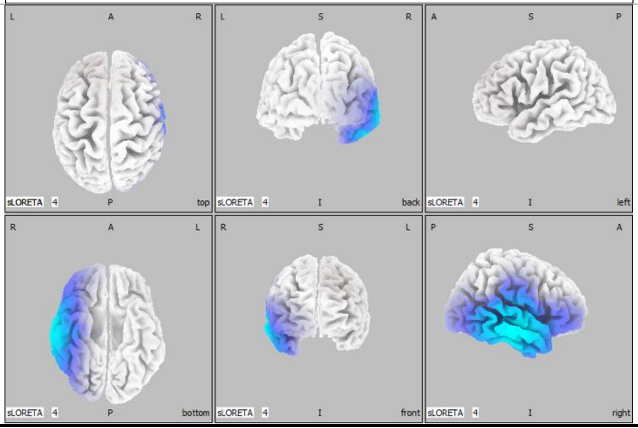

		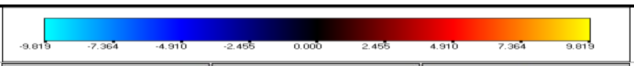
Subject13	Value= -2,77E-1<br>(X= 55 , Y= 35 , Z= 0) (MNI coords)<br><br>Brodmann area 47<br>Inferior Frontal Gyrus<br>Frontal Lobe	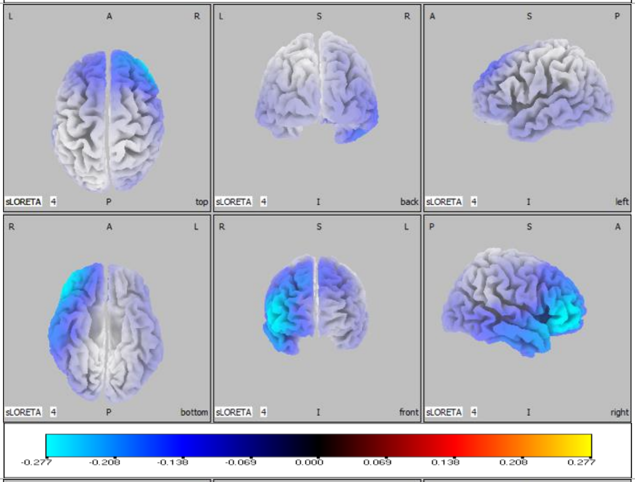
Subject14	Value= -1,80E+0<br>(X= -20 , Y= 65 , Z= -10) (MNI coords)<br><br>Brodmann area 11<br>Superior Frontal Gyrus<br>Frontal Lobe	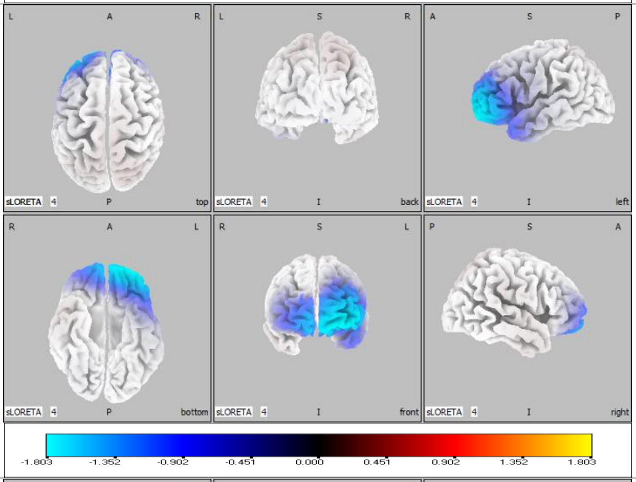
Subject15	Value= 1,02E+0<br>(X= -45 , Y= -55 , Z= 55) (MNI coords)<br><br>Brodmann area 40<br>Inferior Parietal Lobule<br>Parietal Lobe	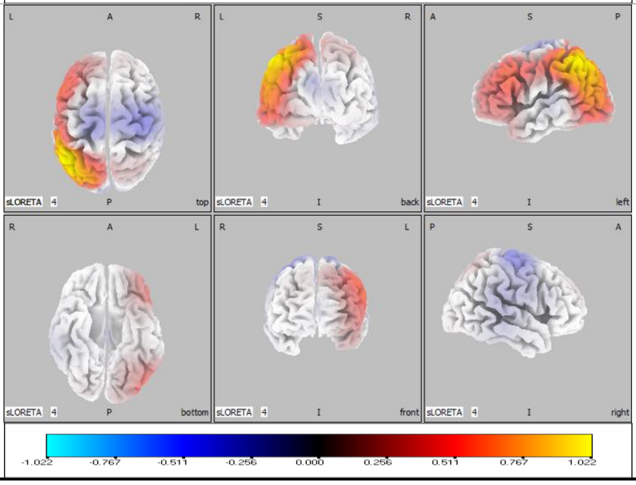

Subject16	Value= 1,46E+0<br>(X= 45 , Y= 55 , Z= -5) (MNI coords)<br><br>Brodmann area 10<br>Middle Frontal Gyrus<br>Frontal Lobe	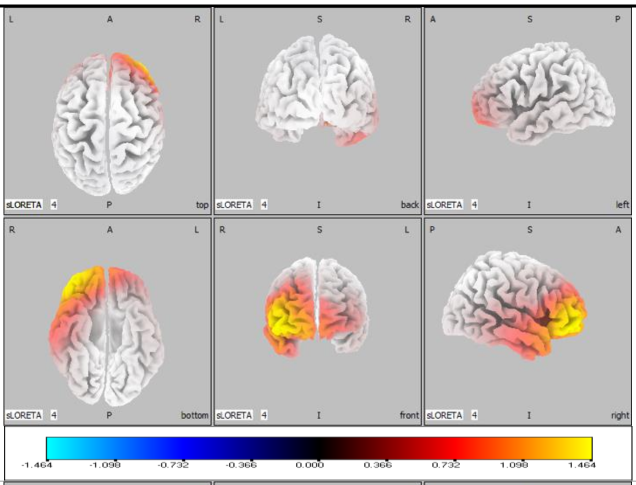
Subject17	Value= 9,93E-1<br>(X= 35 , Y= 60 , Z= -10) (MNI coords)<br><br>Brodmann area 10<br>Middle Frontal Gyrus<br>Frontal Lobe	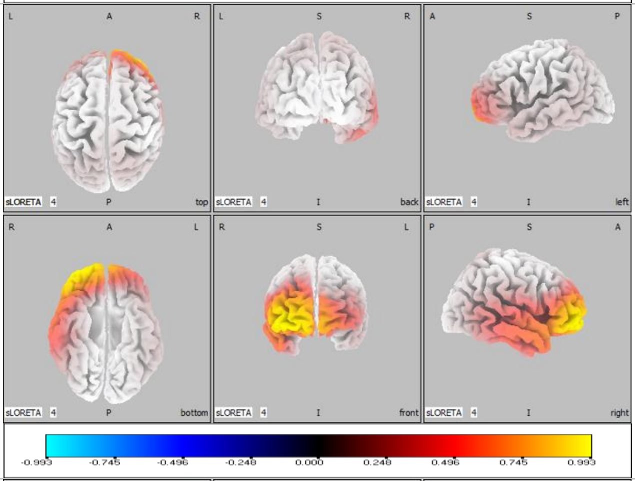
Subject18	Value= 2,55E+0<br>(X= 25 , Y= 50 , Z= 40) (MNI coords)<br><br>Brodmann area 9<br>Superior Frontal Gyrus<br>Frontal Lobe	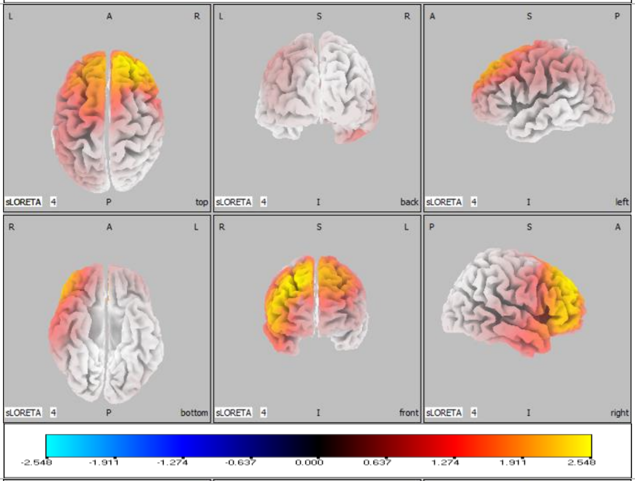
Subject19	Value= 1,15E+0<br>(X= 65 , Y= -5 , Z= -20) (MNI coords)<br><br>Brodmann area 21<br>Inferior Temporal Gyrus<br>Temporal Lobe	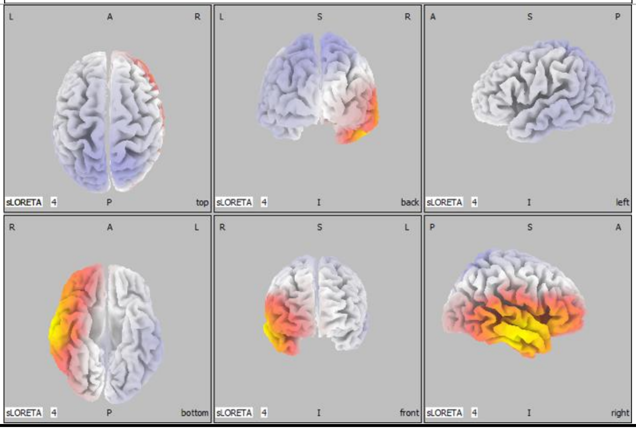

		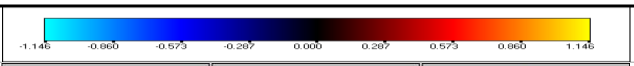
Subject20	Value= 3,07E+0<br>(X= -30 , Y= 50 , Z= 35) (MNI coords)<br><br>Brodmann area 9<br>Superior Frontal Gyrus<br>Frontal Lobe	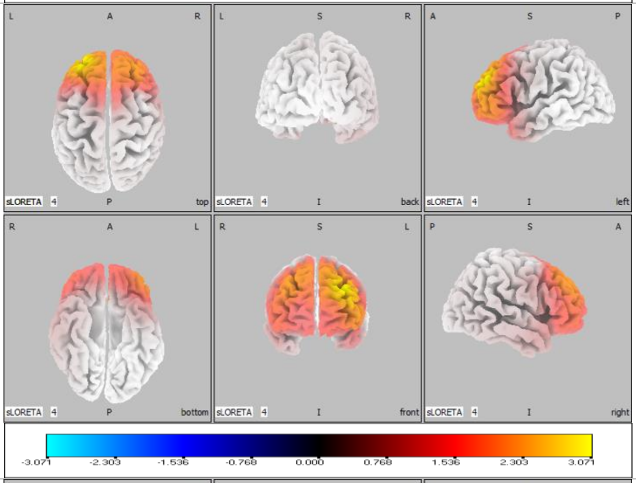
Subject21	Value= -1,02E+0<br>(X= 15 , Y= 65 , Z= -15) (MNI coords)<br><br>Brodmann area 11<br>Superior Frontal Gyrus<br>Frontal Lobe	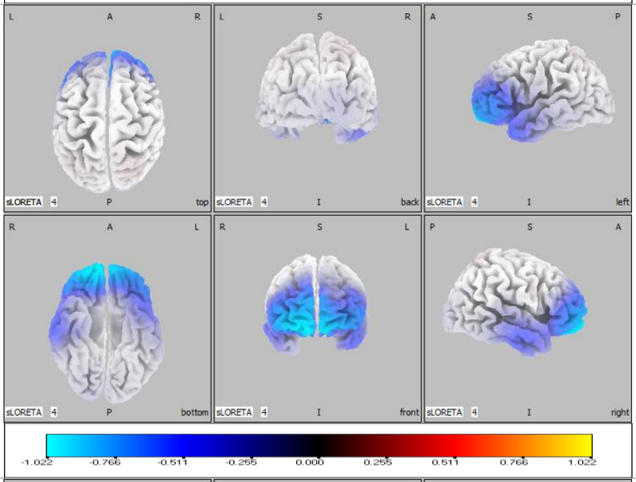
Subject22	Value= -5,24E-1<br>(X= -5 , Y= -100 , Z= 20) (MNI coords)<br><br>Brodmann area 18<br>Cuneus<br>Occipital Lobe	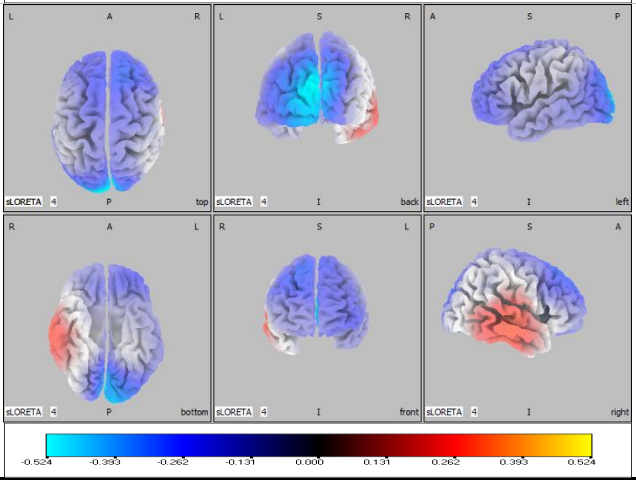

Subject23	Value= 1,20E+0<br>(X= -50 , Y= 45 , Z= -10) (MNI coords)<br><br>Brodmann area 47<br>Inferior Frontal Gyrus<br>Frontal Lobe	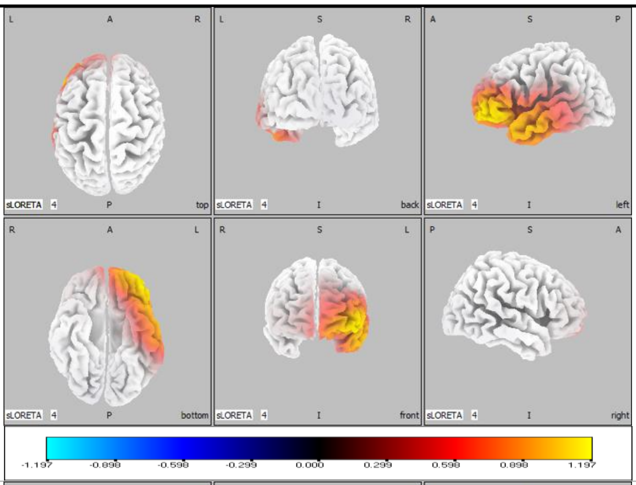
Subject24	Value= 2,86E-1<br>(X= 15 , Y= 65 , Z= -15) (MNI coords)<br><br>Brodmann area 11<br>Superior Frontal Gyrus<br>Frontal Lobe	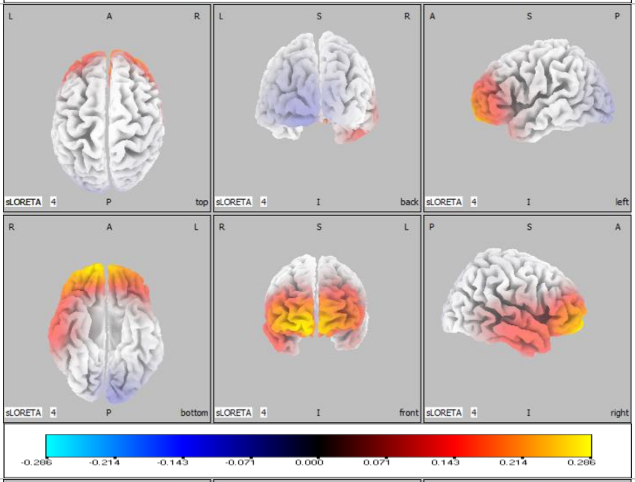
Subject25	Value= 2,73E-1<br>(X= -15 , Y= 65 , Z= -15) (MNI coords)<br><br>Brodmann area 11<br>Superior Frontal Gyrus<br>Frontal Lobe	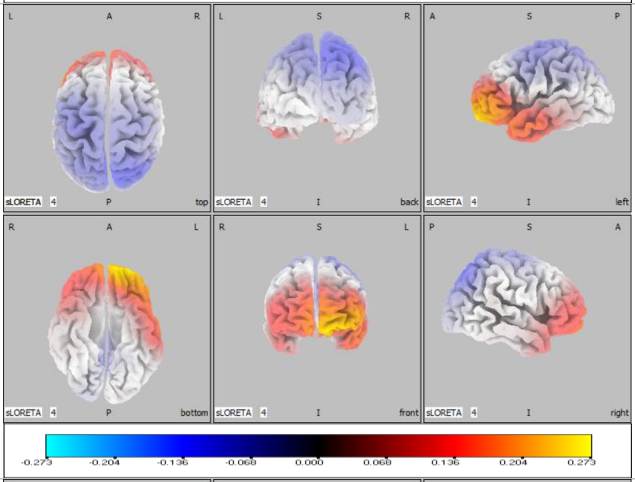
Subject26	Value= 2,21E-1<br>(X= -55 , Y= 30 , Z= -5) (MNI coords)<br><br>Brodmann area 47<br>Inferior Frontal Gyrus<br>Frontal Lobe	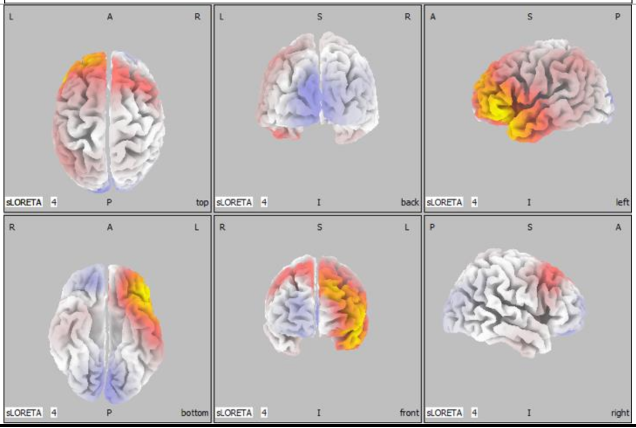

		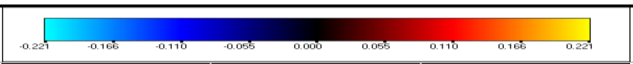
Subject27	Value= 6,67E-1<br>(X= 25 , Y= 60 , Z= -15) (MNI coords)<br><br>Brodmann area 11<br>Superior Frontal Gyrus<br>Frontal Lobe	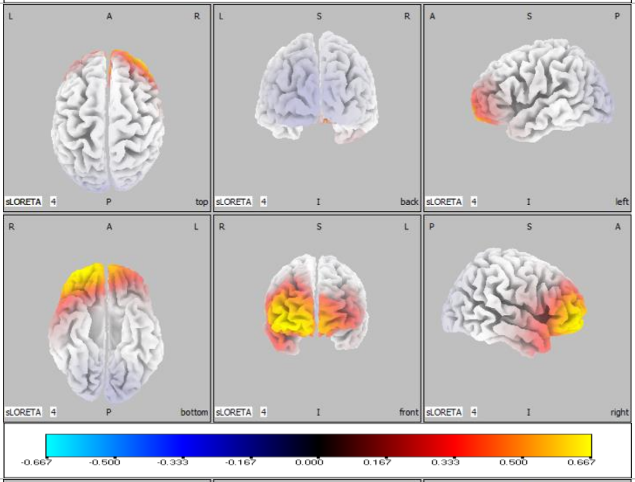
Subject28	Value= 1,08E+1<br>(X= 5 , Y= 65 , Z= -15) (MNI coords)<br><br>Brodmann area 11<br>Medial Frontal Gyrus<br>Frontal Lobe	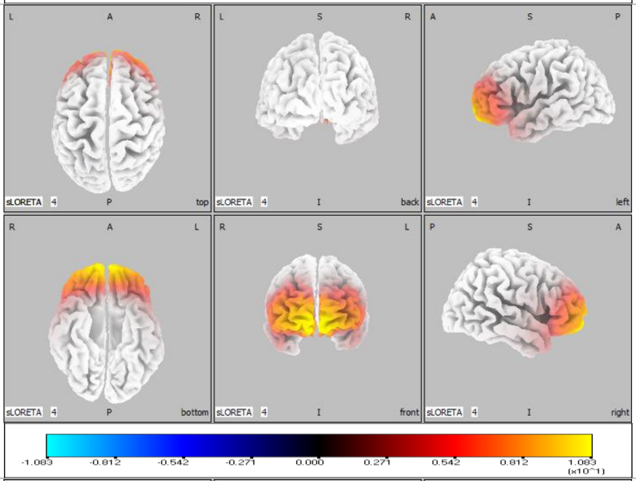
Subject29	Value= 2,02E-1<br>(X= 5 , Y= 65 , Z= -15) (MNI coords)<br><br>Brodmann area 11<br>Medial Frontal Gyrus<br>Frontal Lobe	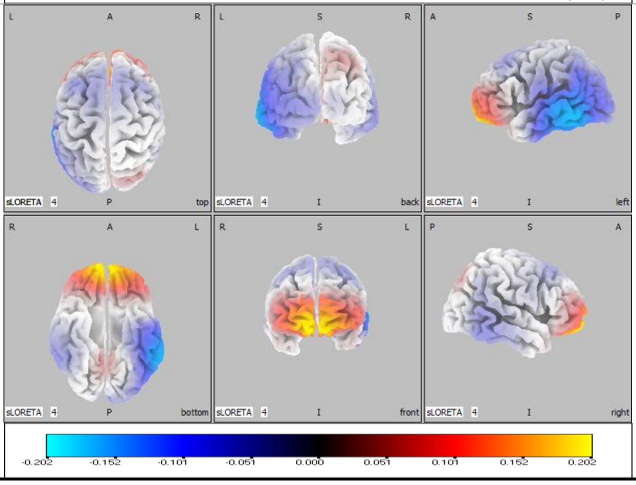

Subject30	Value= 5,00E-1<br>(X= -10 , Y= -60 , Z= 70) (MNI coords)<br><br>Brodmann area 7<br>Postcentral Gyrus<br>Parietal Lobe	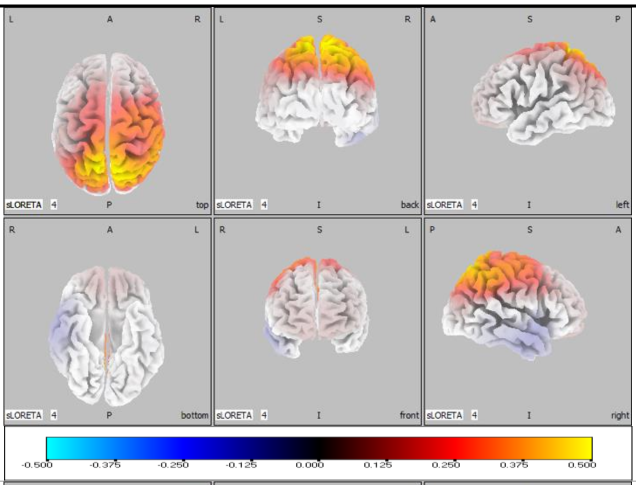
Subject32	Value= 3,17E-1<br>(X= 60 , Y= 0 , Z= -25) (MNI coords)<br><br>Brodmann area 21<br>Middle Temporal Gyrus<br>Temporal Lobe	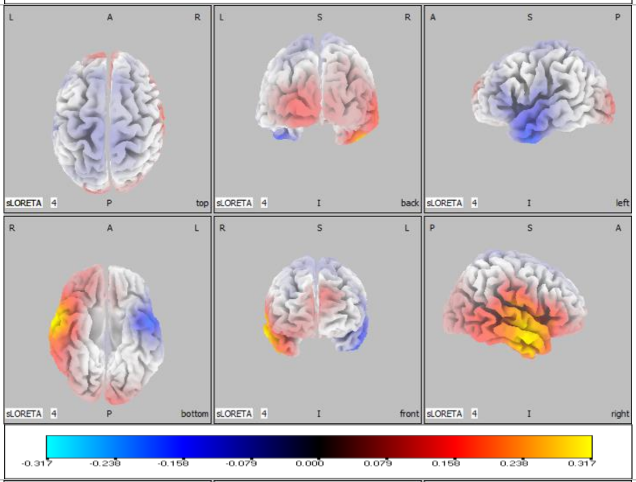
Subject33	Value= -2,46E-1<br>(X= 20 , Y= -95 , Z= 20) (MNI coords)<br><br>Brodmann area 19<br>Cuneus<br>Occipital Lobe	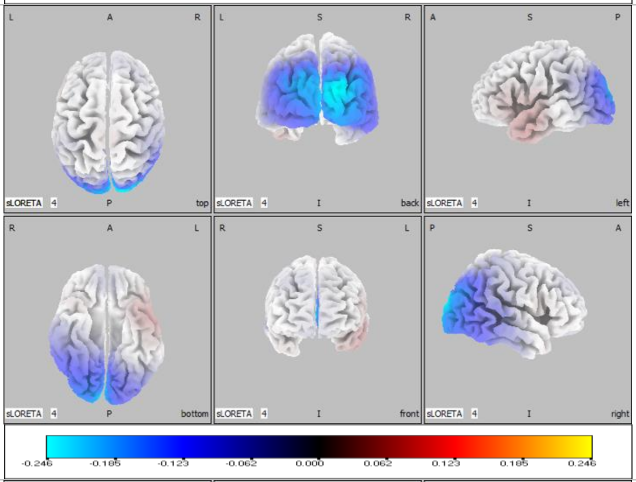
Subject34	Value= 5,59E-1<br>(X= -25 , Y= 55 , Z= 30) (MNI coords)<br><br>Brodmann area 10<br>Superior Frontal Gyrus<br>Frontal Lobe	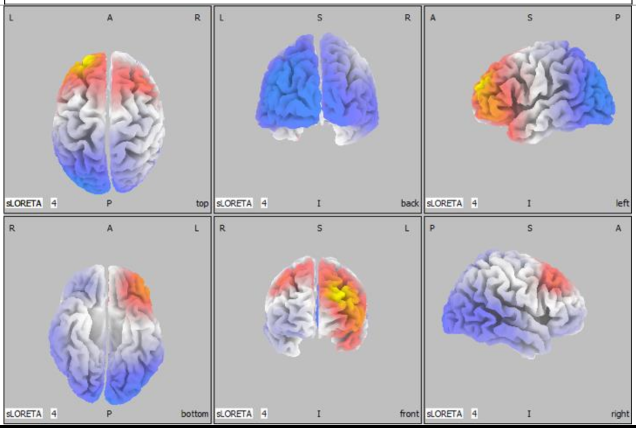

Subject35	Value= 1,08E+0<br>(X= -40 , Y= 55 , Z= -10) (MNI coords)<br><br>Brodmann area 11<br>Middle Frontal Gyrus<br>Frontal Lobe	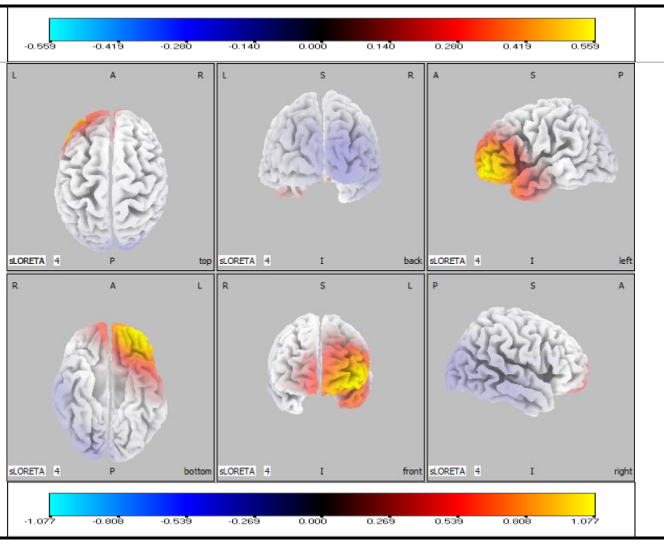
